# The *Environmental Commons* in Urban Communities: The Potential of Place-Based Education

**DOI:** 10.3389/fpsyg.2019.00226

**Published:** 2019-02-08

**Authors:** Constance Flanagan, Erin Gallay, Alisa Pykett, Morgan Smallwood

**Affiliations:** ^1^Department of Civil Society and Community Studies, School of Human Ecology, University of Wisconsin–Madison, Madison, WI, United States; ^2^School of Human Ecology, University of Wisconsin–Madison, Madison, WI, United States

**Keywords:** environmental education, place-based learning, citizen science, youth civic action, environmental commons, urban ecology

## Abstract

The reflections of 205 4–12th graders (most from racial/ethnic minority backgrounds) on what they learned from participating in place-based stewardship education (PBSE) projects in their urban communities were analyzed. All projects involved hands-on collective learning/action by teams of students, teachers, and community partners in the communities where students attended school. Reflections were analyzed using an iterative process of deductive and inductive coding and identifying emergent themes. Deductive coding was informed by the authors’ earlier theoretical and empirical studies on the environmental commons (EC) and the key principles outlined in Elinor Ostrom’s work on effective group practices for stewarding common pool resources. Reflections were coded for up to 8 discrete references to the two elements of the *environmental commons*: (1) the natural resources on which life depends (awareness of nature in the urban space; nature’s diversity and ecological balance; interdependence of humans with nature; healthy environments and species’ well-being; students’ environmental identities; and human impact and agency); and (2) collective actions to protect a community’s resources (benefits and responsibilities of team work; within-group dynamics and civic skills; collective efficacy; generativity; and identification with the broader community). We found that students articulated, with varying levels of understanding, the two key EC elements. Most referred to positive human impact and one-third mentioned negative human impact. When discussing the community benefitting from their work, a majority mentioned humans; yet nearly half referred to other species or living systems; and a quarter referenced generativity, i.e., the legacy of their work for the future. Concerning the collective orientation of projects: one-third felt collective action was imperative for solving environmental issues, half expressed feelings of collective efficacy, and over one-third referenced their increased attachment and identification with a broader community (school, city, or nature). Core practices in this PBSE model parallel the elements of effective groups identified by Ostrom. We conclude with a discussion of the potential of PBSE projects in urban communities for developing young people’s sense of the public realm more broadly and their stake in the natural environment and their communities.

## Introduction

Human impact on the Earth’s natural environment poses major challenges for current and especially future generations. To address these challenges, policies at national and international levels are critical. However, as the political economist, Elinor Ostrom (2012), argued, national and global solutions will only work if people are committed to them at local levels. In this paper, we explore a model of environmental education that emphasizes what young people can do at the local level and examine its role in nurturing their environmental awareness and commitments. Specifically, we assess what 4–12th grade students learn about the *environmental commons* by participating in place-based stewardship education (PBSE) in their urban communities. First, we define PBSE and describe the specific model. Next we discuss the *environmental commons theory* informing our work and review key elements of PBSE as enacted in these urban contexts. Finally, we summarize students’ reflections on what they learned from participating in this PBSE model.

### Place-Based Stewardship Education

Place-based stewardship education refers to experiential education about the natural environment in the local community (Gallay et al., 2016b). The focus on local *place* is two-fold: as a source for learning and as a community to which students can contribute by applying what they learn. In the PBSE model we are assessing students, teachers, and community partners work in teams to define an environmental issue impacting their community, collect and analyze data, and take actions to mitigate the problem. An evaluation of this model with middle-school students in a rural community found significant increases in students’ enjoyment of nature, pro-environmental behavior, community attachment, and confidence in their civic capacities for environmental action (Gallay et al., 2016a). However, youth in rural areas have a different experience of the natural environment. For example, compared to their peers in urban communities, these rural youth spent more time outdoors and enjoyed nature more (Gallay et al., 2016a).

Here we examine the insights of urban students who participated in similar PBSE projects. Many projects take place in racial/ethnic minority communities and thus explore an increasing focus in environmental education (EE) (Bouillion and Gomez, 2001; Barnett et al., 2006; Russ and Krasny, 2015, 2017). Although urban communities of color are disproportionately affected by environmental pollution, until recently, neither the mainstream environmental movement nor the lion’s share of environmental education has concentrated on the experiences of people of color (Taylor, 1996, 2014). Scholars also have criticized the racialized representation of nature in policy and popular culture with African-Americans rarely depicted in natural spaces (Finney, 2014). Notably, when youth of color get engaged in environmental activism it is typically because they see connections between the health of the environment and that of their community and culture (Quiroz-Martinez et al., 2005).

In contrast to a view of nature as a pristine landscape apart from the city, urban environmental education and projects emphasize the interdependent relationships of humans and natural systems in the city and the civic potential of local residents (including children) to assess the quality of the environment and to act to improve it. The PBSE projects discussed here include practices that are common in urban environmental education including the city as classroom, problem-solving, stewardship, youth as assets in community development, and the city as a social-ecological system (Russ and Krasny, 2015). The potential of such projects for children’s development inheres in the combination of environmental learning, direct experiences with nature, and civic actions to mitigate environmental problems and improve the community. In line with a “civic ecology” framework, the projects discussed here emphasize resilience and human agency. They combine the restorative benefits of being in nature with development of the capabilities to observe and improve the natural environment and to see how human behavior and choices (including the students’ own) affect the ecosystem (Krasny and Tidball, 2009).

In documenting the PBSE projects, we have aimed to advance theory about the *environmental commons* which we define as: (1) the natural resources and systems on which life depends, and (2) the public spaces and processes in which people work together to determine how they will care for those resources and for the communities they inhabit (Flanagan et al., 2016; Gallay et al., 2016a,b). In earlier studies, we arrived at this definition through a grounded approach to analyzing students’ reflections regarding what they learned from participating in projects (Gallay et al., 2016a). Our theory also has been informed by the work of Ostrom and colleagues who identified the characteristics of groups that make them effective in sustaining common pool resources (CPRs) such as fisheries or forests that provide benefits to everyone but can be depleted if overused. Characteristics of effective groups include: proximity to the specific CPR; the strength of members’ identification with the team and its goal of sustaining the resource; and dynamics within the group including mutual respect, responsibility and communication over time that enable members to know one another and to build trust (Cardenas and Ostrom, 2006).

Core practices in the PBSE projects we have been studying parallel these elements of effective groups outlined in Ostrom’s work. With respect to proximity, students’ attention is drawn to the natural environment as it operates and is affected by humans in the *local place* where they live and attend school. Ostrom’s second element, identification with the group and its goal, is emphasized via a *collective structure for learning and action* about the natural environment in teams of students, teachers, and adult community partners. Finally, diversity in the experiences and perspectives of team members is considered an asset in the PBSE projects and dynamics within groups emphasize *mutual respect and communication*. Since most teams work over a period of a few months to a year, members should get to know and trust one another.

### Natural Systems in Urban Contexts

The emphasis on local place in these projects means that nature is not treated as a wilderness remote from urban life. Rather, the interdependence of humans with natural systems within the urban ecology is emphasized. Toward that end, two things are needed. First, youth must *notice* nature and natural systems as they operate in and are an integral part of their everyday lives. In addition, they must realize that human behaviors impact the natural environment in positive and negative ways, that people can choose what impact they will have, and that the youth themselves can be agents of positive change.

The latter emphasis on young people’s agency contrasts with the inertia identified in Kahn’s (2002) studies in a Texas bayou which led to his coining the term, *environmental generational amnesia*. Although the youth he interviewed knew in the abstract that pollution is bad for the environment, many did not take notice of the polluted settings they lived in. Kahn theorized that, over time, environmentally degraded settings had become the new normal. The possibility that they might reverse environmental harm seemed not to be within the youths’ purview. By contrast, in the projects we are documenting, students engage in collective action with the goal of mitigating environmental harm. Consequently they should realize that *what is* need not define *what could be*.

The collective action to improve the community’s environment that is at the core of these PBSE projects is a form of civic engagement that may continue into adulthood. National longitudinal studies indicate that opportunities for community contribution and a public voice in adolescence increase the likelihood of civic action (voting and volunteering) in adulthood (McFarland and Thomas, 2006). Identifying with their local community and ways they can contribute to it may be especially empowering for youth in urban areas that have been marginalized from the mainstream. The very fact that they are engaging in collective action to improve the debilitative conditions in their neighborhood may be a means whereby these young people can challenge negative narratives and reclaim their community’s identity (Ginwright and Cammarota, 2007).

## Sites and Projects

All of the projects described in this paper take place in metropolitan areas where over half of the state’s population resides. All projects are school-based and are part of a regional network of school-community partnerships organized by the Southeast Michigan Stewardship Coalition (SEMIS)^1^. The region has been affected by deindustrialization but the combination of economic change, migration, and social dis/investments have had differential effects on the three communities where the students in our sample attend school and engage in projects.

The first site, a former industrial and manufacturing powerhouse, is now a predominantly African-American urban community of concentrated poverty in which abandoned houses and vacant lots dot the landscape. The second site is an ethnically diverse urban community where population and median income are growing but where the poverty rate remains high. Students in these first two communities may live within a few miles of a river or natural area and some may have woods or greenspaces within walking distance. However, many have had little experience with the natural environment. In contrast, students in the third community, with a well-educated middle-class population, have ample and accessible neighborhood parks, natural landscapes, and outdoor recreation.

Students’ projects are responsive to local conditions but share a common set of practices including: ecological observations, data collection and analyses, learning and action in teams of students, teachers, and community partners, and presentation of results in public venues (with students, teachers, and community partners from other schools, city or county administrators, elected officials). The specific content of projects discussed in this paper focus on one of the following: a study of the ecological history of land use in the community and humans’ relationships to their food which led to permaculture practices in growing food on the school grounds; investigation of a local urban park’s ecological and social history, water quality sampling and storm water management leading to flood mitigation through installation of a bioswale; community mapping and investigations which led to studies of local food economies and urban gardens; water quality and habitat health investigations of local water bodies which led to design and creation of water filtration systems; studies of sustainability, biodiversity, local agriculture and food systems, incorporating climate change research, invasive species removal, construction and maintenance of school food garden and harvesting vegetables; community surveys, planning, design and creation of a community park by reclaiming neighborhood abandoned houses and vacant lots; photo-essays of strengths and opportunities in the community’s natural and built environments and community murals of local African American history; and a study of availability and access to healthy, pesticide free food which led to planting and care of an urban community garden.

## Materials and Methods

The study was completed as an evaluation in collaboration with SEMIS, who has permission to conduct programming and evaluation in the schools. The study was reviewed by the University of Wisconsin Education and Social/Behavioral Science Institutional Review Board and the IRB determined that the project is evaluation and does not constitute research as defined in 45 CFR 46.102(d). While parental consent was not required, we have followed ethical considerations in informing participants of the study, obtaining verbal assent, and maintaining the confidentiality of participants.

### Sample

The sample includes 205 4–12th graders (78% high-, 6% middle-, and 16% upper elementary school; 52% male) who participated in a SEMIS project. Students’ race/ethnicity was available for 92% of the sample: 66% identified as African American, 15% European American, 7% Latinx/Hispanic, 6% Multiracial, 3% Asian American, and 3% Arab American. The racial/ethnic composition of the sample is consistent with the demographic characteristics of public schools in the three communities. In two of the communities, just under 75% of students qualify for free or reduced lunch, and 21% qualify in the third community.

### Measures

After completing their projects, a reflective prompt was developed by the study team asking students to: Write a letter to the SEMIS Coalition telling them why you think the work you did in (project specific) was important. What did you learn about your community, other people or species in your community or the environment from the work you did? What did you learn about what kids can do to solve environmental problems in their communities? How has your community or the environment changed because of your work? Students were told that the purpose was not to assess knowledge but to reflect on their experience in their own words. Reflections were written during class time. Students were told that their responses were confidential and would not affect their grades.

### Analyses

Coding of these data was informed by two earlier studies with different samples of students participating in similar PBSE projects. In the first study with a small group of urban students, grounded theory was employed to coding reflections on what students learned and valued about their projects (Strauss and Corbin, 1998). Categories included references to humans’ need for natural resources, interdependence between humans and other living things, depletion and sustaining of resources, negative impact of humans on natural systems, civic learning, skills in civic action and agency, and pride in being community environmental leaders (Gallay and Flanagan, 2016).

In the second study, rural students reflected on what they valued most about their projects. Inductive and deductive methods were used to develop a coding scheme that built on: categories from the first study, research on environmental identity (Clayton, 2003; Jia et al., 2015), and Ostrom’s work on common-pool resources (Cardenas and Ostrom, 2006). Although students in the second study were not asked how their work had affected their community, many discussed the impact on people or nature as something they valued (Gallay et al., 2016a). Categories included references to: the human community (people who would benefit from their work); the environmental commons (benefits to natural systems and non-human species); feelings of attachment to and responsibility for their local community; commitments to continuing environmental monitoring; and generativity (the legacy of their actions on future generations).

For the current project, three of the co-authors began with categories that emerged in the studies cited above, a code-recode process to capture emergent themes, and a final codebook reflecting the two elements of the environmental commons. Codes capturing the first element, (i.e., concepts related to the natural resources and systems on which life depends) included references to: the natural environmental community or commons; the human community or commons; interdependence between humans and other living things and correlations between the health of humans and the quality of natural environments; environmental identity (sense of connection and of care for the natural world); positive and negative human impact. Codes capturing the second element, (i.e., references to people working together to determine how they will attend to and make decisions about their community and its natural resources) included references to: community attachment and pride; generativity; the imperative of collective (not just individual) action; dynamics within their group including civic skills such as communicating, negotiating, finding common ground; feelings of efficacy and agency in effecting change; applying or using scientific and environmental knowledge to address a community need. In addition to these references to some aspect of the Environmental Commons, we coded for references to: individualism, i.e., addressing environmental problems on one’s own; helping that was not directed specifically toward the community or environment; and negative experiences.

Inter-coder agreement was computed (Cohen’s kappa of. 81) and each coder independently coded one-third of the reflections with questions flagged and discussed to reach consensus. Students’ responses were coded for a maximum of 8 categories. For the 205 students, the fifteen codes were applied a total of 944 times with an average of 4.6 codes per student (min = 1, max = 8). After coding, the team conducted a memoing process of all responses connected to a specific code to identify key themes about the Environmental Commons revealed in each code.

## Results

Analyses are organized under the two overarching themes in our definition of the Environmental Commons (EC). The first section (natural resources/systems that support life) summarizes: students’ awareness of nature; its diversity, value, and ecological balance in the urban context; the importance of healthy natural environments to sustain life; the interdependence of humans with other species; students’ environmental identity or sense of personal connection to the natural environment. In the last part of this section we discuss students’ references to human impact (negative and positive) on the environment and awareness of their own agency and resolve to protect natural resources. This last theme – human impact and agency – is a bridge to the second overarching EC theme, i.e., what students learned through the process of collective action in public spaces. Here they discuss the benefits and need for team work, dynamics within their group, and ways that through collective action they have come to have a stake in and identify with a broader community.

### The Environmental Commons: (1) Natural Resources and Systems on Which Life Depends

To appreciate this first dimension of the Environmental Commons, students must become aware of nature and natural systems as part of their urban experience and also understand that the well-being of humans and other species are tied to natural resources.

#### Awareness of Nature

Indications that they were aware of nature included references to “meeting the nature that is right here in our neighborhood,” “what animals’ roles are in our environment,” “that my community has two water systems that come from two different rivers,” and “my community around my school has more wildlife than I thought.” Some noted how they had changed as a result of attending to other living things:

Since I have been in permaculture it had changed me so much I start to like plants and learned new stuff about the plants it showed me how to love plants, if I want to be a permaculture student you should ask about plants and learn about them.

#### Diversity and Ecological Balance

By attending to the details in nature, some students were beginning to notice biodiversity. For example, one pointed to the value of distinguishing plants by their names as a basis for attending to their unique features:

I was able to identify plants. It helps to tell things apart. I used to look at stuff and say, “That’s a plant,” but now I can see differences. It’s important to know names of nature. Things with names have different features.

Others who worked on a community garden noted the needs and functions of different plants and the roles they play in an ecosystem, “that all plants are special in there [*sic*] own special way like trees filter out the air and tomatos need lots of water” and “I learned that some plants will thrive better in more acidic or more basic Ph *[sic]*.” Some projects introduced students to the ecological balance of natural systems and threats to that balance, “I learned how the population of animals go down when the water is bad”; or, alluding to invasive species:

With us removing some of the garlic mustard, along with other invasive plants, other plants had a chance to grow and animals I guess got to eat their food again as invasive species were slowly wiping out the food supply they needed.

A new vocabulary indicating increased ecological awareness was evident in some reflections such as, “The most important thing I learned about our community is how important frogs are to our environment. I also learned that frogs are bioindicators which will tell us if the ecosystem is healthy or not.”

#### Healthy Natural Environments and Species’ Well-Being

Understanding that natural resources support life raises questions about the quality of those resources and an awareness that the well-being of species depends on a healthy ecosystem. In the following reflection on the importance of their water testing project, the student connects the problem of polluted water and land to the survival of different animals:

It was important because it saved the frogs and cleaned the water which is good for the other species and environment too …. And how animals rely on many things to survive and if factories pollute rivers, lakes or any type of land, animals can die off, which can make other animals die off too.

Another student whose group worked on water filtration, observed that water was a resource that sustained life for humans and other animals. “Clean water is important because animals can die from dirty water. It is important for water to be clean because dirty water is not good for your health.” Through the project, her awareness of natural systems in the urban ecology had also changed: “My community/environment has changed because of the filtration project. It has changed the way I see things of water.”

#### Interdependence

Other students referenced the natural systems on which life depends by emphasizing that the well-being of humans and of other living systems were interdependent. Often, that awareness revealed anthropocentric views, i.e., the benefits or utility of nature for humans: “If I clean the water that’s in lakes I can save the species that live in the water such as fish, tadpoles, frogs, snails etc., because those things kill insects and that helps human beings out”; or “I think what we did in permaculture was important because we need plants to ‘survive.”’ Similarly, when discussing the importance of water resources, another student explained that water is:

one of our most vital resources and when our water is polluted it is not only devastating to the environment. This clean, potable water is then used for cooking, drinking, cleaning, bathing, watering our lawns and so forth. We learned that the water/river was very polluted and unhealthy for the residents of this area to swim, drink, or fish in. Also for the wildlife that occupies the river.

Although the reliance of other species on healthy rivers sounds like an afterthought in this quote, some students signaled a more biophilic perspective, i.e., an appreciation, even a love of the natural world – not for its utility for humans, but for its own sake (Wilson, 1984/2003). Some felt that learning about nature increased their sense of connection to and desire to care for it: “I felt it was important to do this because it helps us learn about our eco-system, and feel connected with our Earth.” For another student, learning about plants increased her respect and desire to care for them: “Learning about how plants grow, eat, and live in general, has boosted my appreciation for plants. Learning how plants provide for us and caring for them does great wonders.”

#### Environmental Identity

A feeling of connection to nature has been referred to as an environmental identity (Clayton, 2003) and is associated with time spent in nature (Chawla, 1999) and education about the environment (Ernst and Theimer, 2011) in childhood. Not surprisingly, an environmental identity is correlated with caring for nature (Schultz, 2001; Arnocky et al., 2007; Bamberg and Moser, 2007). Some students commented that their relationship to nature had changed as a result of their PBSE projects: “The walk through ‘River Park’ has been so wonderful. I never cared about nature but now I do. We did so much thinking, gathering ideas with ways we can make the park better (River Park).” Use of the possessive pronoun in the following reflections (e.g., our river, our watershed) indicated both an awareness that life-sustaining resources belonged to everyone and a collective responsibility to care for those resources: “I’ve learned that if we continue to do harmful things to our river, we will regret it in the future” and “if we litter that will end up being in our watershed.” In some cases, students interpreted their new awareness of and relationship with nature as a moral imperative that should impact behavior:

The environment is a wonderful thing that must be treasured and taken care of and not be abused. We should remind or selfs [*sic*] and future generations to not abuse this power and love the environment for all it does for us.

#### Human Impact and Agency

Perhaps because they were both learning and acting, students became aware not only that human behavior affects the environment but also that they have agency and can choose how to act: “I learned that things could be cleaner in our town and that its up to us to fix it”; “We just learned how to give back to our environment, instead of taking it and destroying it”; “The work I did in learning about community gardens was important because it was brought to my attention how much of a food desert our town is and how creating something like a community garden can combat it”; and “by understanding nature we can solve problems and search for more sustainable ways of living.”

In some instances, awareness of human impact was very personal and signaled a resolve to change their ways: “I learned how I have been polluting for years in ways I didn’t know.” References to changes in everyday behavior were common. After working on a community garden project a student explained, “Participating in this opened our eyes to other things we could improve on in our daily lives to further help the environment. From food, to transportation, and at home energy consumption”; while another stated, “This project was amazing! It lead me to watch the foods my family and I intake as well as allowing me to become aware of the corrupt processing placed [*sic*] just to have a home-cooked meal.” In some cases, students resolved to redress the negative impacts of others. For example, “people damage our water by throwing things into the water. So people like us have to know how to clean the water people have damaged.” Ultimately, stewardship of the environment implies a need for constant vigilance to maintain healthy ecologies as captured in the following reflection from a student who worked on a bioswale, “The things we did in the community. I check up on it every time I leave school. The community is doing good people basements [*sic*] are not flooding no more.”

### The Environmental Commons: (2) Collective Action in Public Spaces

Students’ reflections also were coded for references to collective action in public spaces, the second Environmental Commons (EC) theme, i.e., the public spaces and processes in which people work together and make decisions about how they will care for the resources they share and the communities they inhabit. It bears repeating that projects take place in local public settings – schools, abandoned lots, parks, rivers. Students’ attention might be drawn to actions they could take to mitigate flooding in their school’s baseball field, to growing food in a school garden, or to lobbying the city to remove abandoned houses and working with residents to turn the space into a public park.

Besides the public settings of projects, the PBSE model emphasizes collective learning/action in teams of students, teachers, and community partners. In analyzing their reflections on this dimension of the Environmental Commons, we were interested in students’ perceptions of the benefits of team work and whether any students felt that they could do this work on their own; their insights into group dynamics and what skills or capabilities they gained from being part of the team; and how, through their civic contributions, they developed an identification with and helped to define the broader community (the school they attended or town where they resided) and the public or common good.

#### Benefits of and Need for Team Work

Although our coding scheme included a category for individualism, i.e., doing work on one’s own, no responses were assigned to this category. In contrast, many students said they learned that teams were more effective than were individuals for achieving their project goals: “you get more done with a team than by yourself”; and “we can solve problems if we work together.” With environmental restoration a goal, one student felt that collective action was imperative: “We found out we need a group to bring back the environment we once had twenty years ago.” Another noted the benefits of multiple perspectives and ideas for solving public problems: “doing this filter project was a great chance to see what doing something together can get you. I also learned that with teamwork and everyone’s brain working together you can accomplish anything”; and “from this experience, I learned that if students work together and ‘brain storm’ they can create and do just about anything.” Some concluded that working together is not only beneficial, but also enjoyable, “with them being there it was more fun and interesting to express everyone’s ideas.”

#### Dynamics Within the Group

According to Ostrom, groups that are effective in stewarding environmental resources prioritize the group’s goal over individual goals and gain trust by communicating and getting to know one another. In their own words, students captured similar group dynamics:

I think this gardening project was important to gain knowledge and trust. After this project I feel closer more connected to my class. We didn’t work individual. We worked as a team. What I learned through this project is that my class isn’t just a class were [*sic*] a family.

Trust involves giving others the benefit of the doubt (Flanagan and Stout, 2010) and the following student observed how his trust in team members developed through the experience of hearing their different approaches to problem solving: “I learn that everybody has their own ways to learn and figer [*sic*] out, ways to solve problems. I learn to always trust my teams no matter what.” Another mentioned the patience that collective work demands and, ultimately, the satisfaction of a team effort:

It was very tricky and irritating but at the end I really didn’t want the project to end. I also learned that you have to deal with all different kinds of attitude while in a group. I think that when the students come together to actually work, they come up with good work.

#### Civic Dispositions and Skills Gained Through Team Work

Besides trust, students alluded to the enlarged sense of community they had gained through projects. Recall that the PBSE projects are done by teams of students, teachers, and adult partners from the community, working together on behalf of the environment. Due to their intergenerational character and exposure to community organizations, the projects have unique potential for expanding youths’ community networks and developing their social capital, opportunities that are missing in the lives of many young people (Hart et al., 2008).

Like adults who work with youth in community service (CS) projects, the adult partners in these projects tend to be people with strong commitments to the common good. Working alongside such dedicated adults should enlarge the sense of community and boost the social trust of the young participants. Studies of CS show that, compared to other kinds of extracurricular activities, participation is associated with more affirmative views of a community’s intergenerational relations and of people in general (Flanagan et al., 2014).

Similar positive views about the people and organizations in their communities were invoked in the following students’ reflections: “What I learned about the community is that we have a lot of helpful and meaningful people all around us. They don’t mind helping us out either.” In the face of challenges, students’ commitments were buoyed when they realized they weren’t the only ones working on the issue: “there are a few organizations that have already implemented their strategies to combat the food desert in our town” and “I learned that my community had a lot of these invasive species, but I also learned that there were people who cared a lot about that woods and spent a lot of time helping.”

Other skills that students felt they gained are civic competencies that facilitate public problem-solving including communication, perspective taking, tolerance, and an identification with and sense of belonging to the team. One learned that “kids can solve environmental problems, that they can come together and listen to ideas and think of ways to make change and make everyone feel involved.” In order to find common ground, students also have to listen to different perspectives and work through disagreements. One student learned that people are all different and that “sometimes you have to work with people we don’t agree with” while another noted the need to “communicate with others even when there is a problem.” Communication learned within groups was a skill carried over to students’ interactions beyond their team: “I have learned how to understand, help, and communicate with people in school as well as total strangers.”

#### Collective Efficacy

Students were proud of and felt a sense of efficacy from their work. When expressing what they learned about what kids can do to solve environmental problems, many emphasized the power of youth: “I learned that we can and will do everything that an adult can do” and “I learned that age doesn’t matter.” When discussing why projects were effective, some referred to the collective nature of the work: “I learned how local grown food can help the community prosper and that it takes team effort to run such a big garden”; “My work with ‘River Park’ has helped me learn that with the help of my peers, I could make a difference in my community. Kids have much more power than they think.”

At times, students enjoyed public recognition for the mark they were making in their community, as captured in the following account of a student whose group was reclaiming abandoned land: “We were outside and somebody driving past said, ‘I like what you all are doing to this community. Before there were boarded up houses.”’ Other students wondered if their actions might have a ripple effect:

The work we have done is important because it’s a way to help our community. When a community does not care, it reflects when you look at the community. This is also true when a community does care. This work makes everyone more positive and can inspire people to go out and continue the work of others.

Positive public recognition of their work enabled youth to reframe the narrative about who they are and what their school or community could be. Referring to the experience of their team presenting their project at a community forum, one student felt that presenting in that public venue “helped the way people look at us. Everyone thinks our town or school is just so bad and full of uneducated kids but we think different.” Another commented: “My community has changed a lot because now they are starting to see we students, teenagers, and children mean business and we are going to be successful.”

#### Generativity

Not only did some members of the public change their beliefs about the youth but some of the young people themselves alluded to the new self-image they were developing, including the sense that their work could leave a legacy for those who would come after them: “it also made me think about the world in a different/better way that could help future generations”; “the work that we did at [River Park] was very important because we need to protect our environment. The environment that we live in needs to be preserved for our future generation.”

#### Identifying With the Broader Community

Making a palpable contribution to the communities where they live and learn is a core element of these projects and several students described how they came to identify with and have a stake in their communities as a result. References to “*our* school” or “*my* community” were common: “Over the 2 years I worked on these projects, my community has changed physically. Our school use to look like a back alley and now its beautiful.” A student who worked on a community mural, felt that the work was “important because I felt like I finally made a mark in my community.” In the following quote, another student notes how his attitude toward the broader community changed because work on the environmental team taught him to “be in” and be responsible for his community:

It was important for me to work and be a member of the “Eco Team” because it helped me believe in my community. At first I really didn’t care about my surrounding but working with the “Eco-Team” made me realize I need to do something about it. Also my community changed a lot because it looked better than it was before *I started being in the community* (italics added).

### Summary

We have emphasized themes relevant to an understanding of the Environmental Commons that emerged in our coding. Here we summarize the percentages of students’ responses that referenced each code. A majority (76%) referred to positive human impact whereas only 32% noted ways that people negatively affect the environment. The stronger emphasis on positive behaviors may reflect the proactive nature of these projects and the sense of agency students feel, which is also affirmed by the fact that 51% alluded to feelings of empowerment and efficacy. Although we coded for negative statements (boring, don’t like being outdoors), only 5 students said anything negative about their projects.

When discussing the *community or commons* that would benefit from their work, a majority of the statements referred to people (57%) although 42% mentioned other species or natural systems as part of the community and 21% specifically referenced the interdependence of healthy natural systems and human well-being.

Students also alluded to a sense of connection or attachment to a broader community gained through their projects:15% of the reflections referenced sensitivity to the natural environment and 35% a sense of attachment and pride felt as a member of the community at school or in the city. The imperative and benefits of working as a team to solve environmental problems were noted in 34% of the reflections and 12% specifically cited the civic skills (e.g., communication, trust) and dynamics that made their group effective. Finally, 25% suggested that the environmental contributions that they and others were making could leave a legacy by having a positive impact on the natural environment that future generations would inherit.

## Discussion

In their own words, these students from urban communities articulated, with varying levels of understanding, the two key elements of the environmental commons: awareness of natural resources that support life and the processes whereby humans can sustain those resources through collective action. Concerning the first element, students referenced their experiences with the natural world, sometimes noting how they had not previously noticed nature or its diversity and now attached greater value to the natural world – the rivers, the fish, the plants, the “environment” more broadly. Some used possessive pronouns (e.g., our river, our earth, our water, our ecosystem, our environment) alluding to the Environmental Commons principle that the resources that sustain life belong to everyone.

With rare exceptions, students’ descriptions were positive and pointed to their increasing affinity for the natural world. As they paid attention to nature, became familiar with the names of plants, got to know the needs, functions, and roles of various species and the connections between humans and those species, some expressed an ethic of care for the natural world and a responsibility to sustain it for future generations.

Awareness of the natural world in their urban space was coupled in many reflections with references to the impact of human behavior (and often their own) on the natural world. Importantly, references to negative human impact did not often result in cynicism or pessimism. Perhaps due to the emphasis on collective action in this PBSE model, the negative impact of humans was seen as a choice and was met with students’ resolve to turn things around, including, for some, changing personal behaviors. Students said that their eyes were opened to things they could improve and that, “people like us have to know how to clean the water people have damaged.” Human agency and personal resolve were a far more common reaction than passivity and acceptance of the status quo. In some cases, students’ dawning awareness of the finite nature of natural resources and of the impact of human choices sparked what others have referred to as “generative concern,” i.e., a sense of unrest or worry about how one’s actions in the present might affect the conditions of life in the future (Jia et al., 2015).

According to national trend studies, adolescents in the United States are more likely to engage in environmental conservation behaviors if they are aware that some natural resources are finite and that technology may not provide an easy fix (Wray-Lake et al., 2010). Thus, the combination of building awareness of the fragile natural environment in the urban ecology and learning what people can do to sustain and protect it as outlined in the PBSE model presented in this paper is a win-win.

A meta-analysis of research on pro-environmental behavior indicates that awareness of one’s interdependence with other people and species motivates actions to protect that larger community (Bamberg and Moser, 2007). Through their projects, some students felt that they had gained an understanding of the interdependence of humans with the natural world. This awareness may be a foundation for moral responsibility for the natural environment. According to Bandura (2007) one of the ways people morally disengage from environmental responsibility is to disregard or demean the recipients (whether human or other living things) of their actions. To combat this ignorance, it is important that people understand that their actions impact other living things. The fact that 42% of the reflections referred to non-human species as beneficiaries of the environmental projects suggests that students are developing a regard for these recipients of their actions.

With respect to the second dimension of the Environmental Commons, through their collective actions with fellow community members – from their own and other generations – students develop a stake in the local environment and community and an awareness of their own capacity to act. Consistent with an action competence model of environmental education, knowledge is combined with action in these projects (Jensen and Schnack, 1997). The emphasis on problem solving should help younger generations cope with the environmental challenges they will inevitably face armed with ideas, resolve, and a language of possibility (Jensen and Schnack, 1997; Mogensen and Schnack, 2010). The collective action emphasis in these PBSE projects is somewhat unique in education but may be a valuable tool for curbing pessimism in light of the scale of problems such as climate change. To cope with climate change, leveraging the social context in which people make decisions and emphasizing the power of the group to effect change are effective strategies (Roser-Renouf et al., 2014).

Statements coded in the second dimension evoke the defining features of the public realm as outlined by the political theorist, Hannah Arendt (1958). For example, students’ sense of identification with the broader community and pride in their team’s civic contribution echo Arendt’s argument that the public realm is the common world that gathers people together, that it represents a diversity of experiences and perspectives, and that activity in that realm helps people to realize their personal stake in the common good. Arendt (1958) also points out that people will bring a diversity of experiences and standpoints to discussions and actions in this realm. Indications that students were developing an impression of this aspect of the public realm were revealed in comments about the value of different perspectives (“everyone’s brain working together”) for group problem solving and how trust developed as members of the group listened to one another’s ideas about how to achieve their shared goal. Because they partnered with adults from the community, students also were exposed to different generations of people active in the public realm. References to meeting people and getting to know organizations that were “helpful” and “cared a lot” point to the success of these projects in expanding youths’ social networks and developing their social capital (Hart et al., 2008).

In some projects students worked to get abandoned properties in blighted neighborhoods removed and then to replace the empty spaces with public parks. Such projects are examples of what scholars have called *critical civic praxis* in which urban youth of color exercise collective agency to change debilitative neighborhood conditions (Ginwright and Cammarota, 2007). Through such praxis, students asserted their right to beautiful public spaces where they and fellow residents can gather. They also assumed responsibility for their communities, and, by making their mark, showed that they cared about and believed in those communities. Not only had they reclaimed public space but they also rewrote the narrative about themselves and their communities.

Recognition of students’ critical civic praxis from fellow members of the community echoes another characteristic of work in the public realm, as outlined by Arendt (1958), i.e., it is seen and heard by everyone. When residents of the community witnessed the students’ work and thanked them, students realized that they were transforming the public’s image of young people, that, as one young person put it, “they are starting to see we mean business and are going to be successful.” The fact that their positive public contributions can be seen and heard by everyone holds particular promise in the more blighted communities where stereotypes and prejudice persist. The resolve to change those stereotypes was captured in a student’s observation that their project, “helped the way people look at us. Everyone thinks our town or school is just so bad and full of uneducated kids but we think different.”

The projects documented here also challenge dominant representations of humans in the natural environment (in advertising, consumption, and outdoor recreation) as people who are White and middle-class. As Finney (2014) suggests in her book, *Black Faces, White Spaces,* such images constrain our imagination and our beliefs about who has knowledge and who cares about nature. In fact, contrary to these stereotypical images, many contemporary environmental justice organizations are led by youth of color who apply an intersectional analysis linking race, class, and the natural environment to address the root causes of environmental injustice and offer hopeful solutions (Quiroz-Martinez et al., 2005; Gallay et al., 2016a). In addition, the focus on nature in urban areas opens new possibilities for human awareness and vigilance about protecting the natural environment, an awareness that is critical as urban communities deal with the challenges of climate change.

### Limitations and Future Directions

The limitations in our study were based, in part, on our goal of building theory grounded in a particular model of PBSE in urban communities. We relied on students’ own words about what they learned from their practice and thus our work is emergent. Because relatively little is known about how youth in these communities experience and perceive the natural environment, hearing from them first-hand is a necessary foundation for constructing valid instruments (Karabenick et al., 2007). That said, claims about change in students’ environmental understanding are limited by the fact that data were not collected prior to their engagement in the PBSE projects. Future studies should establish a baseline of students’ understanding of the natural environment as experienced in their urban context before they participate in projects and then assess change associated with participation. In addition, insofar as students apply subject matter (science, math, etc.) to address a local environmental issue, future work should explore whether their academic interests are piqued by making a meaningful contribution to their communities and whether they see the affordances of learning environmental science for realizing community goals.

## Conclusion

Students’ reflections on what they learned from engaging in these PBSE projects resonate with the elements of groups that make them effective in managing common-pool resources: proximity to the issue and immersion in work in the local context, identification with the shared goals of the group, and the trust and respect that stem from engagement with the group over time (Cardenas and Ostrom, 2006). Our focus on youth and the environmental commons brings the elements that Ostrom identified into the field of youth civic development. Whereas Ostrom focused on the qualities of effective groups for preserving common-pool resources, the field of youth civic development examines the practices and relationships through which young people develop the skills, dispositions, and motivations that foster life-long civic commitments.

Scholars of youth civic development have pointed to the civic-science nexus as a rich context where younger generations can gain skills they will need to grapple with 21st century challenges (Hart and Youniss, 2018). Likewise, a new model of ecological citizenship suggests that children’s stake in environmental justice and civic decision-making should be simultaneously nurtured through practices that combine collective agency, deliberation, and self-transcendence (Hayward, 2012). Perhaps the most urgent issue of the 21st century is how people will adapt to changes in the Earth’s natural environment that humans have created. Educating younger generations about human interdependence with nature and nurturing a sense of vigilance about that delicate balance is critical. However, knowledge of the issues and motivation to do something must be balanced with collective actions in communities where young people can gain both a sense of agency and the reassurance that they do not have to solve the problems on their own.

## Author Contributions

CF was the PI on the project and took the lead on the conceptualization and writing of the manuscript. EG took the lead on data collection while EG, AP, and MS coded and analyzed the data and provided feedback on the manuscript.

## Conflict of Interest Statement

The authors declare that the research was conducted in the absence of any commercial or financial relationships that could be construed as a potential conflict of interest.

## Footnotes

1 Organizational website is http://semiscoalition.org/. SEMIS is one of nine regional hubs of the Great Lakes Stewardship Initiative (GLSI) which engages schools and community partners in PBSE.
